# Effect of Left Ventricular Outflow Tract Obstruction on Left Atrial Mechanics in Hypertrophic Cardiomyopathy

**DOI:** 10.1155/2015/481245

**Published:** 2015-12-16

**Authors:** Lynne K. Williams, Raymond H. Chan, Shemy Carasso, Miranda Durand, Jimmy Misurka, Andrew M. Crean, Anthony Ralph-Edwards, Christiane Gruner, Anna Woo, John R. Lesser, Barry J. Maron, Martin S. Maron, Harry Rakowski

**Affiliations:** ^1^Division of Cardiology, Toronto General Hospital, Toronto, ON, Canada M5G 2C4; ^2^Department of Cardiology, Papworth Hospital NHS Foundation Trust, Papworth Everard, Cambridge CB23 3RE, UK; ^3^Department of Cardiology, Baruch Padeh Medical Center, Poriya and Bar-Ilan University, 15208 Tiberias, Israel; ^4^Department of Medical Imaging, Toronto General Hospital, Toronto, ON, Canada M5G 2C4; ^5^Department of Cardiac Surgery, Toronto General Hospital, Toronto, ON, Canada M5G 2C4; ^6^Division of Cardiology, University Hospital Zurich, 8091 Zurich, Switzerland; ^7^Hypertrophic Cardiomyopathy Center, Minneapolis Heart Institute Foundation, Minneapolis, MN 55407, USA; ^8^Hypertrophic Cardiomyopathy Center, Tufts Medical Center, Boston, MA 02111, USA

## Abstract

Left atrial (LA) volumes are known to be increased in hypertrophic cardiomyopathy (HCM) and are a predictor of adverse outcome. In addition, LA function is impaired and is presumed to be due to left ventricular (LV) diastolic dysfunction as a result of hypertrophy and myocardial fibrosis. In the current study, we assess the incremental effect of outflow tract obstruction (and concomitant mitral regurgitation) on LA function as assessed by LA strain. Patients with HCM (50 obstructive, 50 nonobstructive) were compared to 50 normal controls. A subset of obstructive patients who had undergone septal myectomy was also studied. Utilising feature-tracking software applied to cardiovascular magnetic resonance images, LA volumes and functional parameters were calculated. LA volumes were significantly elevated and LA ejection fraction and strain were significantly reduced in patients with HCM compared with controls and were significantly more affected in patients with obstruction. LA volumes and function were significantly improved after septal myectomy. LVOT obstruction and mitral regurgitation appear to further impair LA mechanics. Septal myectomy results in a significant reduction in LA volumes, paralleled by an improvement in function.

## 1. Introduction

Hypertrophic cardiomyopathy (HCM) is an inherited cardiac disorder characterized by pathological left ventricular (LV) hypertrophy, complex pathophysiology, and diverse clinical outcomes. Increased LV mass and diastolic dysfunction are associated with progressive left atrial (LA) dilatation and dysfunction, often compounded by the presence of LV outflow tract (LVOT) obstruction and concomitant mitral regurgitation. LA size and volume have been shown to be determinants of both exercise capacity [1] and major adverse cardiac and cerebrovascular events in patients with HCM [2–4]. In addition, LA dysfunction and in particular LA booster pump function have been shown to correlate with heart failure symptoms in HCM [5] as well as being a strong predictor for the development of atrial fibrillation (AF) requiring hospitalization [6]. A recent study of a large cohort of HCM patients undergoing CMR has demonstrated LA ejection fraction (LAEF) and minimum LA volumes as predictors of the development of AF [7].

The relationship between the LV and LA is highly dynamic and interdependent. All phasic aspects of LA function are to some degree affected not only by LV contractility, relaxation, compliance, and filling pressures, but in addition by intrinsic LA contractility, relaxation, and compliance [8, 9].

**Figure 1:**
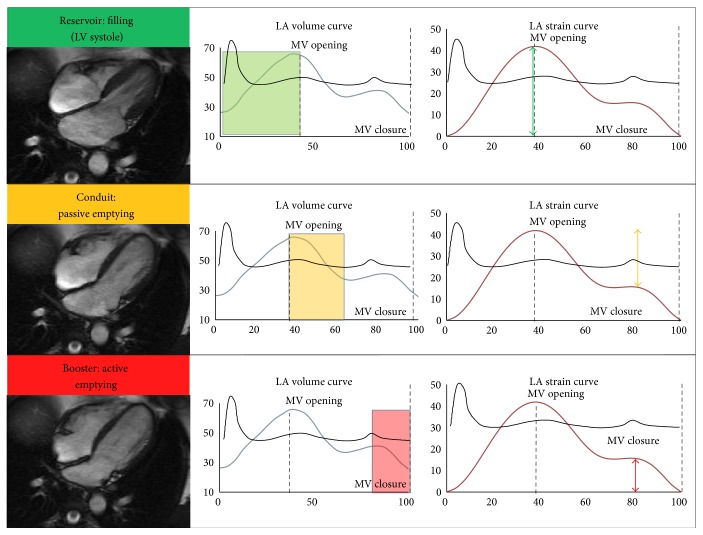
Diagrammatic representation of phasic LA function, LA volume, and strain curves. An ECG curve is superimposed to highlight timing of LV systole and diastole.

LA strain and strain rate analysis by means of feature tracking provides a feasible noninvasive method of assessment of LA function [10–12]. Three distinct phases of atrial function assessed are (1) reservoir function, which represents the storage of pulmonary venous return during ventricular systole and isovolumic relaxation, (2) conduit function, which represents the period of passive emptying of the LA down a pressure gradient during early diastole, and (3) booster or active contractile function, which represents intrinsic atrial contractility during which the atria empty before the end of ventricular diastole (see Figure 1). A recent study has demonstrated the feasibility and reliability of quantification of LA strain and strain rate using CMR myocardial feature tracking in both normal controls and patients with HCM [13].

The aim of this study was to evaluate alterations in LA volumes and function in patients with HCM, particularly between patients with and without LVOT obstruction, and to identify the determinants of LA myopathy in this disease state. In addition, we aimed to study the effects of relief of LVOT obstruction on LA function in a subset of patients undergoing septal myectomy.

## 2. Methods and Materials

### 2.1. Study Population

A retrospective cohort of one hundred adult patients from the Hypertrophic Cardiomyopathy Clinic at the Toronto General Hospital with HCM (maximal septal thickness of  ≥15 mm and a septal-to-posterior wall thickness ratio of ≥1.3, in the absence of another cardiac or systemic disease that could cause LV hypertrophy) was included in the study. All patients had preserved LV systolic function (defined as a CMR-derived LVEF ≥55%). Patients were in sinus rhythm at the time of both the CMR study and echocardiogram. Studies were performed on active cardiac medications. Patients were subdivided into two groups as follows: (1) a nonobstructive HCM subgroup with LVOT gradient of <30 mmHg both at rest and with provocation (with Valsalva and amyl nitrate) and (2) an obstructive HCM subgroup with resting LVOT gradient of ≥30 mmHg. Patients with latent obstruction only were not included in the current study. A cohort of fifty normal controls who had previously undergone CMR at the Tufts Medical Center and the Minneapolis Heart Institute were included for comparison. In addition, a subset of twenty patients with obstructive HCM who had undergone CMR studies before and after septal myectomy was studied.

### 2.2. Clinical and 2D Echocardiographic Data

A retrospective chart review was performed in order to obtain demographic data and symptomatic status. Standard 2D echocardiographic data was obtained from the study performed closest to the CMR, with all echocardiographic measurements acquired as per ASE guidelines [14, 15]. Mitral regurgitation was qualitatively assessed by a single observer and graded as none, trivial, mild, moderate, or severe. The study was approved by the Research Ethics Board of the Toronto General Hospital and the Investigational Review Board of the participating centers in the United States of America.

### 2.3. CMR Protocol

At the Toronto General Hospital, CMR imaging was performed on 1.5T or 3T whole body magnets (Magnetom Avanto, Magnetom Verio, Siemens Healthcare, Erlangen, Germany) using a 32-element phased-array coil. At Tufts Medical Center, CMR imaging was performed on a Philips Gyroscan ACS-NT 1.5T scanner (Best, Netherlands) and at the Minneapolis Heart Institute on a Siemens Avanto 1.5T scanner (Erlangen, Germany). Cine steady state free precession (SSFP) images were acquired in short axis (sequential 10 mm slices from the atrioventricular ring to the apex) and 2-, 3-, and 4-chamber long axes. LV ejection fraction, ventricular volumes, ventricular mass, and maximal wall thickness were measured by standard offline analysis using customized software (QMassMR, Medis, Leiden, Netherlands).

### 2.4. MR Velocity Vector Imaging

VVI is a feature-tracking method which incorporates feature and endocardial contour tracking. VVI quantifies myocardial motion by automatically tracking user-defined endocardial and epicardial contours to define the inward and outward myocardial motion. Based on motion of the tracked points between the frames and knowledge of the time interval between frames, 2D tissue velocity is computed. Strain and strain rate are computed by the range in the relative distance between localized tracked trace points, combined with the difference in the relative displacement of the tissue motion between tracked points. Strain was defined as the instantaneous local trace lengthening/shortening and strain rate as the rate of lengthening/shortening.

The feature-tracking program, VVI Version 3.0.0 (Siemens Healthcare, Mountain View, CA), was applied to the cine SSFP images from archived studies, allowing for strain parameter assessment. Cine SSFP data derived from CMR images were converted from Digital Imaging and Communications in Medicine (DICOM) to Audio Video Interleave (AVI) format creating 30 cardiac phases. Subsequently, LA motion was quantified by automatic tracking of user-defined points in both the subendocardial and subepicardial regions.

### 2.5. Left Atrial Volumes

LA volumes were determined by VVI software from the four-chamber view using Simpson's method of disks. Pulmonary veins and the LA appendage were excluded in the calculation of volumes. The following were measured (indexed to body surface area): (1) maximum volume at end-systole (*V*_max_), (2) pre-*A* volume prior to the onset of atrial contraction (*V*pre_*A*_), and (3) minimum volume at end-diastole (*V*_min_). From these volumes, LA phasic parameters were derived as shown in Table 1.

### 2.6. Left Atrial Mechanics

In the long-axis four-chamber views, endocardial and epicardial borders were manually traced in the end-systolic frame. The software subsequently traced the borders in the other frames of the cardiac cycle automatically. Strain parameters were recorded after visual confirmation of the best endocardial and epicardial motion tracking (by operator subjective visual assessment). The strain curves were gated in systole (*R* wave), and longitudinal strain/strain rate parameters were calculated. A diagrammatic representation of the phases of LA function is shown in Figure 1.

### 2.7. Intra- and Interobserver Variability

Offline analysis of all cine SSFP data sets was performed by a single observer (LW). Ten randomly selected studies were reanalyzed by the same observer (LW) and a second observer (JM).

### 2.8. Statistical Analyses

Continuous and categorical data are expressed as mean (±SD) or *n* (%), respectively. Comparison between the HCM subgroups and normal controls was performed using an ANOVA. Intra- and interobserver variability were assessed using Bland-Altman analysis. Correlations between variables were assessed using Pearson's correlation or Spearman's rank correlation coefficient test where appropriate. The independent effects of LVOT obstruction on LA strain parameters were tested using multivariable linear regression models. All statistical analyses were performed using SAS 9.3 (Cary, North Carolina) and MedCalc version 11.6.0.0 (MedCalc Software, Belgium). Statistical significance was defined as a 2-sided *p* value <0.05.

## 3. Results

### 3.1. Patient Demographics and Clinical Data

#### 3.1.1. Clinical, Demographic, and 2D Echocardiographic Parameters

Baseline demographic and echocardiographic parameters for the normal control group and the HCM group as a whole are shown in Table 2, with the data for obstructive and nonobstructive HCM subgroups shown in Table 3.

#### 3.1.2. Cardiovascular Magnetic Resonance Imaging

Conventional CMR parameters are shown in Tables 2 and 3. While LVEF and LVEDVi were not significantly different between HCM patients and normal controls, LVMI was significantly elevated in patients with HCM (80.6 ± 26 versus 52.5 ± 11 g/m^2^; *p* < 0.0001). There was no significant difference in LVEDVi between HCM patients with and without obstruction. However, patients with obstructive HCM had a higher LVMI (87.7 ± 24 versus 73.0 ± 26 g/m^2^; *p* = 0.006).

### 3.2. LA Volumes and Myocardial Mechanics

**Figure 2:**
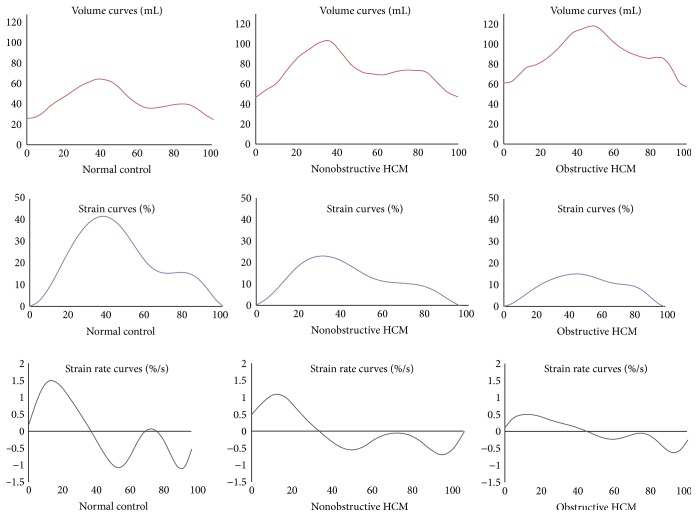
Representative volume, strain, and strain rate curves from a normal control and patients with nonobstructive and obstructive HCM.

All LA volumes were significantly elevated in patients with HCM, and LAEF significantly reduced (44.8 ± 9 versus 65 ± 11%; *p* < 0.0001), with all phases of LA function (reservoir, conduit, and booster) affected. These findings remained even after adjustment for age, LVMI, and LVEDVi. Representative LA volume, strain, and strain rate curves are shown in Figure 2.

### 3.3. Relationship between LVOT Obstruction and LA Mechanics

Patients with obstructive HCM had a significantly greater impairment in LA function and larger LA volumes (Table 4), and LAEF was significantly lower (42.3 ± 8 versus 47.2 ± 9%; *p* = 0.004). Although conduit function was not different between the two HCM subgroups, patients with obstruction had significantly more impairment of LA reservoir function, with lower reservoir strain (14.5 ± 4 versus 17.7 ± 5%; *p* = 0.002) and strain rate (0.59 ± 0.2 versus 0.73 ± 0.2%/s; *p* = 0.001). In addition, there was a significant reduction in both booster strain (6.1 ± 2 versus 7.5 ± 3%; *p* = 0.01) and strain rate (−0.44 ± 0.1 versus −0.58 ± 0.25/s; *p* = 0.004). The reduction in LA function and increase in LA volumes remained significant even after adjusting for age, LVMI, LVEDVi, and degree of mitral regurgitation.

### 3.4. Correlation of LA Mechanics and Echocardiographic and CMR Parameters

**Figure 3:**
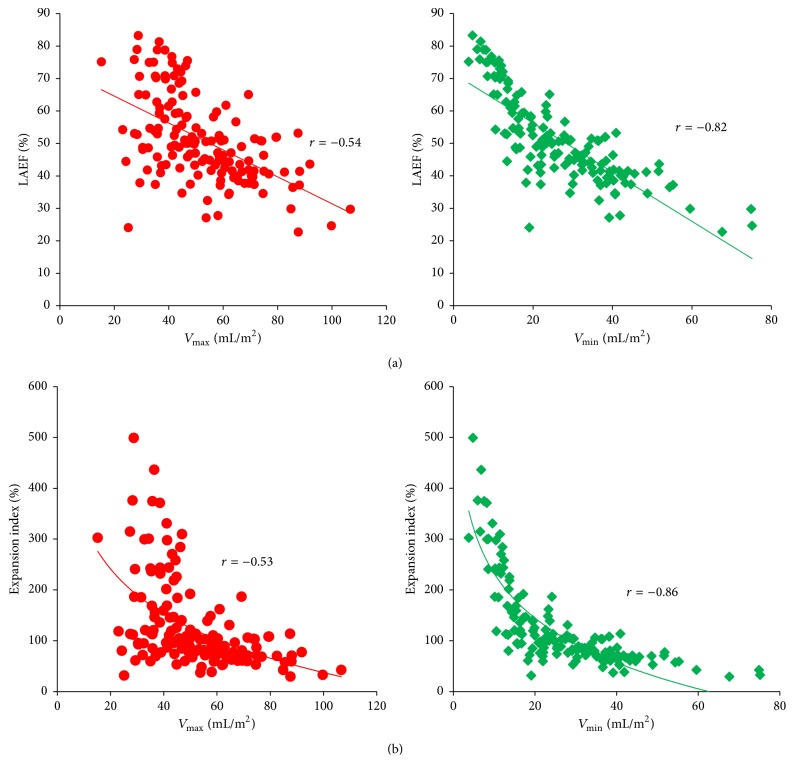
(a) Correlation between LA ejection fraction (LAEF %) and maximum/minimum LA volumes. (b) Correlation between LA expansion index and maximum/minimum LA volumes.

There were no significant correlations between strain or strain rate parameters and 2D echocardiographic parameters of diastolic function or between resting/provocable LVOT gradients and either strain or strain rate parameters. LVMI correlated inversely with reservoir strain (*r* = −0.61; *p* < 0.0001), booster strain (*r* = −0.52; *p* < 0.0001), and LAEF (−0.56; *p* < 0.0001). Correlations between LA volumes and LA mechanics are shown in Figure 3 and Table 5.

### 3.5. Effect of Septal Myectomy on Left Atrial Volumes and Mechanics

The mean time from surgery to repeat CMR was 8.6 months (range: 5 to 18 months). No patients underwent a concomitant surgical MAZE procedure. Significant reductions in NYHA class, LVOT gradient, and degree of mitral regurgitation were seen after myectomy (Table 6). LA volumes decreased and LAEF increased significantly (41.6 ± 13 versus 48.4 ± 10%; *p* = 0.006). Although conduit function remained unchanged, an improvement in both reservoir (14.1 ± 6 versus 17.3 ± 7%; *p* = 0.01) and booster strain (6.8 ± 4 versus 9.8 ± 5%; 0.0001) was seen.

### 3.6. Intra- and Interobserver Variability

Intra- and interobserver variability demonstrated good agreement for both volumetric and strain parameters and are shown in Table 7.

## 4. Discussion

In the present study, we demonstrate not only a significant increase in LA volumes but also a marked impairment in all components of LA function in patients with HCM compared with normal controls. While previous studies have demonstrated abnormalities in LA function in patients with HCM, for the first time we have demonstrated significantly worse LA function in those with resting LVOT obstruction compared to patients with nonobstructive HCM. Septal myectomy, via relief of LVOT obstruction and reduction in degree of mitral regurgitation, results in both a significant reduction in LA volumes and improvement in LA function.

The pathological hypertrophy characteristic of HCM, myocardial ischemia secondary to abnormalities of the microvasculature, and the presence of myocardial disarray and fibrosis all serve to result in a reduction in LV compliance, abnormal ventricular relaxation, and diastolic dysfunction. The resultant elevation in LV filling pressures is transmitted back to the LA, necessitating an increase in LA pressures in order to maintain adequate diastolic filling. Subsequent increases in LA wall tension serve to drive LA enlargement, reflected in the significantly greater LA volumes seen in patients with HCM compared with normal controls.

In the present study, patients with LVOT obstruction were older and more symptomatic and had significantly greater LA volumes. In addition, they had evidence of a higher burden of hypertrophy, a greater degree of mitral regurgitation, and more severe diastolic dysfunction. LVMI was shown to correlate inversely with measures of LA strain, but no correlations were noted between absolute LVOT gradient and parameters of strain and strain rate. However, even after multivariate analysis (adjusting for age, gender, LVMI, LVEDVi, and degree of mitral regurgitation), there remained a significant difference in both LA volumes and function between patients with and without LVOT obstruction.

LA reservoir function reflects both active left ventricular contraction (and systolic descent of the mitral annulus) and passive LA stretch and is largely determined both by LV systolic function and by LA compliance. Given that LV longitudinal strain has been shown to be reduced in patients with HCM despite a preserved LV ejection fraction, with an even greater degree of impairment in patients with LVOT obstruction [16], this may in part explain the differences in reservoir function seen in patients with obstructive versus nonobstructive HCM. In addition, the presence of a preexisting atrial myopathy or even fibrosis in the atrial wall may lead to an increase in LA stiffness and a concomitant reduction in compliance. LA chamber stiffness (assessed by invasively measured LA pressure-volume relations) has been demonstrated to be increased in patients with HCM compared with controls and to correlate with the degree of LV hypertrophy [17].

Conduit function appears to be governed mainly by LV relaxation, which is affected by increased LV mass, myocardial ischemia, and the presence of myocardial fibrosis. No significant difference was noted between patients with obstructive and nonobstructive HCM in conduit volume, passive empting index, or SRe, despite a higher LVMI and higher grade of diastolic dysfunction in patients with obstruction.

LA booster function is dependent on preload (via the Frank-Starling mechanism), afterload, and intrinsic LA contractility. While there is initially a compensatory increase in LA contractility in response to an increase in LA volumes, eventually further increases in LA volumes will result in a decline in atrial function, as evidenced by the inverse correlation we have demonstrated between *V*_max_ and LA ejection fraction, reservoir, and booster strain. Sanada et al. have previously demonstrated the effect of LA afterload mismatch on LA booster pump function in HCM [18]. The increase in LA afterload seen in patients with obstruction is likely to contribute to the greater reduction in LA booster strain seen in these patients and may explain the improvement in function seen after septal myectomy.

An intrinsic atrial myopathy may in addition affect active* LA* contraction and booster strain. Indirect evidence to suggest the presence of an intrinsic myopathy in HCM is the increase in the number of calcium antagonist receptors demonstrated in the atrial myocardium of patients with HCM, suggesting an abnormality in calcium fluxes through voltage-sensitive calcium channels that may play a role in atrial dysfunction [19].

In the present study, no correlations were noted between 2D echocardiographic parameters of diastolic function and strain or strain rate parameters. Importantly, no correlation was seen between *A* wave velocity on transmitral Doppler filling profiles and any parameters of LA booster function, suggesting that peak *A* wave may not accurately reflect LA contractile function but rather reflects the atrioventricular pressure gradient between the atrium and ventricle. While LA volumes and transmitral *A* wave are readily obtained from standard transthoracic echocardiography, our present findings highlight the need for more sophisticated techniques for the assessment of LA function if we are to use this information to predict risk of adverse events.

While strain analysis is not yet part of routine clinical practice, the simple addition of measurement of *V*_min_ to routinely measured *V*_max_ appears to provide valuable information regarding function. *V*_min_ demonstrates a stronger correlation with not only measures of global LA function and compliance, but also markers of LA contractility (active and booster function). The present data suggest that measurement of minimum volume provides a valuable surrogate marker of LA function.

## 5. Study Limitations

Although measurements using VVI applied to CMR images have not been previously validated against VVI applied to echocardiographic images in the same patient group for the analysis of LA function, a recent study utilizing the feature-tracking software has demonstrated both the feasibility and reliability of this technique for assessing LA strain and strain rate [13]. Although differences in signal characteristics between different scanners and scans performed at different field strengths might also impact feature tracking, Kowallick et al. have demonstrated good reproducibility of measurements of LA function irrespective of scanner type [13]. The thin-walled LA may result in technical difficulties with the application of feature tracking using VVI. In addition, the LA appendage and origin of the pulmonary veins pose additional challenges to tracking. LA function has previously been demonstrated to be abnormal in patients with primary severe mitral regurgitation without HCM [20], and in the current study patients with obstructive HCM had a significantly greater degree of mitral regurgitation than the nonobstructive group. Some of the effects on left atrial volume and function after septal myectomy may be explained by the relief of mitral regurgitation in addition to relief of outflow tract obstruction. Left atrial fibrosis, which may represent an important intrinsic determinant of left atrial function, was not assessed on CMR in the current study but may account for some of the demonstrated changes in function noted.

## 6. Conclusions

Left atrial volumes and functional parameters are abnormal in patients with HCM and are significantly worse in those with obstruction. The associated effects of left ventricular outflow tract obstruction and mitral regurgitation appear to further impair left atrial mechanics. Surgical myectomy, via relief of obstruction, appears to have a positive impact on both left atrial volume and function, and further studies are needed to assess whether aggressive management of LVOT obstruction will result in a reduction in the occurrence of adverse events, particularly atrial fibrillation.

## Figures and Tables

**Table 1:** Phasic LA function parameters.

*Reservoir function*	
Expansion index (%)	(*V*_max⁡_ − *V*_min⁡_)/*V*_min⁡_ × 100
Left atrial ejection/emptying fraction (%)	(*V*_max⁡_ − *V*_min⁡_)/*V*_max⁡_ × 100
*Conduit function*	
Passive emptying index/fraction (%)	(*V*_max⁡_ − *V*pre_*A*_)/*V*_max⁡_ × 100
Conduit volume (mL/m^2^)	LV stroke volume − (*V*_max⁡_ − *V*_min⁡_)
*Booster/pump function*	
Active emptying index/fraction (%)	(*V*pre_*A*_ − *V*_min⁡_)/*V*pre_*A*_ × 100

*V*_max⁡_: maximal left atrial volume; *V*_min⁡_: minimum left atrial volume; *V*pre_*A*_: left atrial volume immediately prior to atrial contraction.

**Table 2:** Baseline clinical and CMR parameters.

	Normal controls (*n* = 50)	HCM (*n* = 100)	*p* value
*Clinical characteristics*			
Age at CMR (years)	42.6 ± 16	49.7 ± 15	*0.007*
Sex (male, *n*, %)	28 (56%)	70 (70%)	0.09
BSA	1.94 ± 0.3	1.94 ± 0.2	0.96
NYHA I/II/III/IV (%)	100/0/0/0	56/19/25/0	*<0.0001*

*CMR parameters*			
LVEF (%)	62.5 ± 6	63.1 ± 7	0.58
LVEDVi (mL/m^2^)	87.7 ± 16	86.8 ± 13	0.72
LVMi (g/m^2^)	52.5 ± 11	80.6 ± 26	*<0.0001*

CMR: cardiac magnetic resonance; BSA: body surface area; NYHA: New York Heart Association functional class; LVEF: left ventricular ejection fraction; LVEDVi: indexed left ventricular end-diastolic volume; LVMi: indexed left ventricular mass.

**Table 3:** Baseline clinical, CMR, and 2D echocardiographic parameters in the nonobstructive and obstructive HCM subgroups.

	Nonobstructive HCM (*n* = 50)	Obstructive HCM (*n* = 50)	*p* value
*Clinical characteristics*			
Age at CMR (years)	44.7 ± 15	54.6 ± 12	*0.001*
Sex (male, *n*, %)	36 (72)	34 (68)	0.83
BSA	1.92 ± 0.2	1.96 ± 0.2	0.35
NYHA I/II/III/IV (%)	82/14/4/0	32/22/46/0	*<0.0001*
Atrial fibrillation (*n*, %)	3 (6)	4 (8)	0.69
Beta-blockers (*n*, %)	22 (44)	42 (84)	*0.0001*
Calcium channel blockers (*n*, %)	5 (10)	4 (8)	0.73
Disopyramide (*n*, %)	0 (0)	38 (76)	*<0.0001*
ACE-i/ARB (*n*, %)	7 (14)	6 (12)	0.77
Amiodarone (*n*, %)	2 (4)	1 (2)	0.56
Coumadin (*n*, %)	4 (8)	2 (4)	0.40
*CMR parameters*			
LVEF (%)	61.7 ± 6	64.6 ± 7	*0.03*
LVEDVi (mL/m^2^)	86.1 ± 15	87.5 ± 12	0.62
LVMi (g/m^2^)	73.0 ± 26	87.7 ± 24	*0.006*
*2D echocardiographic parameters*			
LVOT resting (mmHg)	6.6 ± 2	56.2 ± 28	*<0.0001*
LVOT provocable (mmHg)	12.1 ± 7	82.7 ± 30	*<0.0001*
Maximal wall thickness (mm)	19.1 ± 5	20.7 ± 4	*0.002*
MR: trivial/mild/moderate/severe (%)	74/26/0/0	18/46/28/8	*<0.0001*
*E* wave (m/sec)	0.68 ± 0.2	0.86 ± 0.2	*<0.001*
*A* wave (m/sec)	0.49 ± 0.2	0.73 ± 0.2	*<0.001*
*E*/*A* ratio	1.54 ± 0.6	1.32 ± 0.6	0.07
Deceleration time (ms)	217 ± 48	264 ± 62	*<0.001*
Isovolumic relaxation time (ms)	89 ± 17	94 ± 23	0.32
*E*/*E*′ ratio	7.2 ± 3	11.2 ± 5	*<0.0001*

CMR: cardiac magnetic resonance; BSA: body surface area; NYHA: New York Heart Association functional class; LVEF: left ventricular ejection fraction; LVEDVi: indexed left ventricular end-diastolic volume; LVMi: indexed left ventricular mass; LVOT: left ventricular outflow tract gradient; MR: mitral regurgitation.

**Table 4:** LA volumes and myocardial mechanics.

	Normal controls (*n* = 50)	Nonobstructive HCM (*n* = 50)	Obstructive HCM (*n* = 50)
*LA volume indices*			
*V*_max⁡_ (mL/m^2^)	38.3 ± 10	54.3 ± 15^*∗*^	63.4 ± 17^*∗*#^
*V*pre_*A*_ (mL/m^2^)	22.3 ± 8	40.1 ± 13^*∗*^	49.3 ± 14^*∗*#^
*V*_min⁡_ (mL/m^2^)	13.3 ± 5	28.9 ± 10^*∗*^	36.9 ± 13^*∗*#^
Left atrial ejection/emptying fraction (%)	65.0 ± 11	47.2 ± 9^*∗*^	42.3 ± 8^*∗*#^
Left ventricular stroke volume (mL/m^2^)	54.5 ± 9	52.6 ± 11	55.5 ± 9
Conduit volume	58.4 ± 26	52.3 ± 22	56.4 ± 23
*Reservoir function*			
Expansion index (%)	219.0 ± 113	95.2 ± 36^*∗*^	76.3 ± 23^*∗*#^
Longitudinal reservoir strain (%)	39.5 ± 13	17.7 ± 5^*∗*^	14.5 ± 4^*∗*#^
SRs (%/sec)	1.37 ± 0.4	0.73 ± 0.2^*∗*^	0.59 ± 0.2^*∗*^
*Conduit function*			
Passive emptying index/fraction (%)	41.6 ± 13	26.4 ± 9^*∗*^	22.3 ± 7^*∗*^
SRe (%/sec)	−0.99 ± 0.5	−0.43 ± 0.2^*∗*^	−0.35 ± 0.2^*∗*^
*Booster function*			
Active emptying index/fraction (%)	39.9 ± 14	28.0 ± 10^*∗*^	25.7 ± 8^*∗*^
Longitudinal booster strain (%)	15.3 ± 7	7.5 ± 3^*∗*^	6.1 ± 2^*∗*#^
SRa (%/sec)	−1.05 ± 0.5	−0.58 ± 0.2^*∗*^	−0.44 ± 0.1^*∗*#^

SRs: strain rate in systole; SRe: strain rate during passive emptying; SRa: strain rate during active emptying; ^*∗*^ *p* < 0.05 compared with normal controls; ^#^ *p* < 0.05 between obstructive and nonobstructive HCM.

**Table 5:** Correlations between LA volumes and myocardial mechanics.

	* V*_max⁡_ (mL/m^2^)		* V*_min⁡_ (mL/m^2^)	
	Correlation coefficient	*p* value	Correlation coefficient	*p* value
Left atrial ejection/emptying fraction (%)	−0.54	<0.0001	−0.82	<0.0001
Expansion index (%)	−0.53	<0.0001	−0.86	<0.0001
Reservoir strain (ST_*S*_, %)	−0.49	<0.0001	−0.73	<0.0001
Booster strain (ST_*A*_, %)	−0.32	0.0001	−0.52	<0.0001
Active emptying index/fraction (%)	−0.35	<0.0001	−0.57	<0.0001

**Table 6:** Effects of septal myectomy on LA volumes and mechanics.

	Premyectomy (*n* = 20)	Postmyectomy (*n* = 20)	*p* value
NYHA I/II/III/IV (%)	0/0/100/0	80/20/0/0	*<0.0001* ^*∗*^
LVOT resting (mmHg)	68.1 ± 40	11.0 ± 6	*<0.0001* ^*∗*^
LVOT provocable (mmHg)	98.3 ± 27	21.2 ± 14	*<0.0001* ^*∗*^
MR: trivial/mild/moderate/severe (%)	10/40/40/10	40/60/0/0	*0.0004* ^*∗*^
LVEF (%)	65.9 ± 8	61.4 ± 9	0.08
*LA volume indices*			
*V*_max⁡_ (mL/m^2^)	66.2 ± 23	49.8 ± 20	*<0.0001* ^*∗*^
*V*pre_*A*_ (mL/m^2^)	53.2 ± 24	39.3 ± 18	*<0.0001* ^*∗*^
*V*_min⁡_ (mL/m^2^)	40.5 ± 23	26.6 ± 16	*<0.0001* ^*∗*^
Left atrial ejection/emptying fraction (%)	41.6 ± 13	48.4 ± 10	*0.006* ^*∗*^
Left ventricular stroke volume (mL/m^2^)	55.5 ± 11	48.0 ± 10	*0.01* ^*∗*^
Conduit volume	57.2 ± 21	47.3 ± 20	0.066
*Reservoir function*			
Expansion index (%)	80.5 ± 44	100.1 ± 36	*0.01* ^*∗*^
Longitudinal reservoir strain (%)	14.1 ± 6	17.3 ± 7	*0.01* ^*∗*^
SRs (%/sec)	0.51 ± 0.2	0.57 ± 0.2	0.15
*Conduit function*			
Passive emptying index/fraction (%)	21.4 ± 9	22.2 ± 9	0.66
SRe (%/sec)	−0.27 ± 0.2	−0.27 ± 0.2	0.98
*Booster function*			
Active emptying index/fraction (%)	26.4 ± 11	33.7 ± 8	*0.002* ^*∗*^
Longitudinal booster strain (%)	6.8 ± 4	9.8 ± 5	*0.0001* ^*∗*^
SRa (%/sec)	−0.48 ± 0.2	−0.68 ± 0.2	*0.001* ^*∗*^

*∗* denotes *p* < 0.05 between pre- and postmyectomy values.

**Table 7:** Inter- and intraobserver variability.

	Diff. means (±1.96 SD)	
	Interobserver	Intraobserver
*V*_max⁡_ (mL/m^2^)	1.5 (±7.7)	1.5 (±2.6)
*V*pre_*A*_ (mL/m^2^)	0.8 (±8.4)	1.5 (±3.0)
*V*_min⁡_ (mL/m^2^)	2.6 (±9.8)	1.5 (±5.8)
Longitudinal reservoir strain (%)	1.3 (±5.2)	0.2 (±6.6)
Longitudinal booster strain (%)	0.5 (±3.1)	0.1 (±3.7)
SRs (%/sec)	0.07 (±0.16)	0.01 (±0.18)
SRe (%/sec)	0.02 (±0.18)	0.00 (±0.37)
SRa (%/sec)	0.09 (±0.26)	0.02 (±0.24)

Difference of means ±1.96 SD: bias and limits of agreement derived from Bland-Altman analysis.
